# Pediatric specific challenges of the single institutional review board mandate

**DOI:** 10.1186/s13063-022-06141-y

**Published:** 2022-03-21

**Authors:** Andrew Hu, Jane L. Holl, Mehul V. Raval

**Affiliations:** 1 Division of Pediatric Surgery, Department of Surgery, Northwestern University Feinberg School of Medicine, Ann & Robert H. Lurie Children’s Hospital of Chicago, 633 N. Saint Clair St, 20th Floor, Chicago, IL 60011 USA; 2 Department of Neurology and Center for Healthcare Delivery Science and Innovation, Biological Sciences Division, University of Chicago, Chicago, IL USA

**Keywords:** Single IRB, Common Rule Revision, Pediatric research, Human participant research

## Abstract

**Background:**

The Common Rule Revision (CRR) mandates a single institutional review board (IRB) for all US federally funded nonexempt multisite human participant research. While the CRR aims to improve research efficiency, its success in pediatric research remains uncertain

**Main body:**

There are multiple challenges that threaten the purported efficiency of the single IRB mandate. While the CRR is clear that ethical review is the purview of the single IRB, responsibility for issues of local study governance are less well defined. Therefore, reliance agreements (RA) must be negotiated between single IRBs and participating institutions. These negotiations can vary significantly based upon the institution’s local context and are often arduous, lengthy, and burdensome. Furthermore, in pediatric research, issues such as assent, surrogate consent, and IRB risk determination add additional layers of complexity that must be considered. No clear system exists for resolving disagreements surrounding these critical human participant protection issues. Finally, the variation in institutional resources directed towards pediatric research may mean that only a select few pediatric institutions will be able to function in the single IRB system. These challenges will need to be overcome to successfully implement the CRR and achieve its objective of improving multisite research efficiency. We suggest that an empiric and collaborative approach utilizing implementation strategies is necessary for the CRR and single IRBs to be effective.

**Conclusion:**

The CRR seeks to improve the efficiency of multisite human participant research in the US. There are multiple challenges that will need to be overcome. An empiric collaborative approach is necessary. If successful, single IRBs have the potential to usher in a new era of impactful and efficient multisite pediatric research.

## Background

The Common Rule Revision (CRR), mandating a single institutional review board (IRB) for all United States (US) federally funded, nonexempt, multisite human participant research, went into effect January 2020 [1]. It represents, arguably, the most significant change to human participant research procedure in the US in decades. Multisite studies previously relied on separate participating institutional IRB review, a process that has been criticized as burdensome, inefficient, and delaying research without benefit [2]. The CRR seeks, by consolidating ethical review to a single IRB, to streamline and accelerate the conduct of multisite research. Efficient multisite review is especially important in pediatric research, where multiple sites are frequently needed to overcome limited study populations. However, empiric data supporting the benefits of single IRBs are scarce and opinion on the mandate is divided [3]. Questions surrounding complex inter-institutional agreements and local contexts raise concerns that the CRR may be trading one imperfect system for another [3]. Universal implementation of single IRBs for pediatric research will be challenging given the diversity of pediatric institutions. Without strategies and resources to support implementation of single IRBs, the success of the CRR in pediatric research remains uncertain.

## Main body

### Reliance agreements

While the CRR clearly specifies that the single IRB is responsible for ethical review, the delineation of roles for local study governance are less clear. These matters, critical to human participant protection, can include investigator qualification, managing conflicts of interest, and data security. Therefore, formal documents, termed reliance agreements (RA), are negotiated between relying sites (participating institutions ceding ethical review) and the single IRB. These RAs clearly assign responsibility, define operating procedures, and provide structure for the collaborative relationship [4]. RAs differ in purpose from clinical site agreements which focus on more financial and legal considerations (e.g., intellectual property rights, insurance, and institutional reimbursement). RA negotiations can vary in complexity as their scope and content depend on the type of study and the priorities of participating institutions. Differences in institutional policies can make a seemingly straightforward task, like drafting consent language, a prolonged series of exchanges, modification requests, and legal reviews [4]. Our own single IRB experience highlighted some of these complexities. In 2019, we launched a multisite trial at 18 pediatric hospitals across the US. The study used a single IRB under a similar 2018 mandate from the National Institute of Health (NIH). Initially, a major problem emerged when none of the participating institutions, including the principal investigator’s own academic pediatric institution, was willing to assume single IRB responsibility. We explored options for engaging a commercial IRB and discovered significant variation in cost with estimates ranging from US $85,000 to $140,000. After retaining an experienced commercial IRB as the single IRB, many participating institutions, nevertheless, still insisted on conducting parallel local reviews of the study protocol. RA negotiations about language in the informed consent documents and use of electronic consents (e-consent) were protracted and challenging, as the individual institutions’ policies for consent and information technology practices varied widely. Communication between the single IRB and participating institutions to resolve these were often repetitive and delayed. As a result, IRB approval of the study was delayed for months and led to significant unanticipated direct costs to the study in addition to onerous administrative burden.

The single IRB related delays and resources used placed the study timeline and goals at substantial risk. Our experience is not unique with similar experiences and concerns being reported by human research protection program (HRPP) staff and other multi-institutional trials [3–8]. Existing pediatric institutional IRBs and organizations were ill-equipped to function within a single IRB system. These challenges may reflect the immaturity of a new system that will improve with time, although this is by no means guaranteed.

Implementing effective RA workflows will require funding and effort to support training, policy adaptations, and organizational restructuring. Yet, the CRR is an unfunded mandate and how well and equally institutions can adapt is unknown. Disparities in effectiveness can only worsen RA complexity as the number and diversity of studies and participating institutions increases. Consequently, the CRR may not eliminate the inefficiencies of duplicative institutional review, but merely transfer them onto the execution of RAs.

### Pediatric local context

A further concern surrounding single IRBs is that by eliminating participating institutions’ IRB review, this will contribute to loss of expertise in relevant local variations in legislation and population characteristics. Knowledge of this local context has the potential to influence the perception of risk and benefits of the intervention studied especially in pediatric research where, unlike adults, ethical consensus can be complex. As children have limited autonomy and need to rely on proxies for consent, a core function of IRBs in pediatric research is to assess participant risk and benefit. Greater than minimal risk requires an anticipated commensurate direct benefit which is subjectively determined by the IRB [9]. However, this determination can be influenced by local contextual factors such as regional socioeconomics, disease prevalence, and cultural/linguistic differences [10]. While institutional IRBs must be qualified to make a risk and benefit determination, such determinations may be influenced by local researchers & HRPP staff experiences and biases (e.g., an IRB that is inexperienced with an intervention may be more conservative in their determinations). Further divergence may occur when agreeing on consent or assent processes. Consistent definitions and applications of assent are often lacking and can vary between institutions. Local IRB decisions may be informed by cultural and religious practices, as well as, state law, for studies involving assent, surrogate consent, emancipated minors, or adolescent confidentiality [11]. For studies in which informed consent is difficult to obtain (e.g., lifesaving interventions in an emergency setting), decisions to allow exemptions of informed consent present a further ethical dilemma and determinations can vary between IRBs [12]. Here, knowledge of the local context is of paramount importance to inform participant risk and benefit but also community consultation and public disclosure [13]. Thus, there are many reasons in pediatric research for institutional and single IRBs to disagree on the acceptability of proposed research. Understandably, institutional IRBs may feel hesitant to relinquish determination of appropriate risk and benefit to an IRB that has little knowledge or understanding of their local contexts, especially as it is local researchers and HRPP staff that will be responsible for explaining the risks and benefits when recruiting participants. Regardless, the CRR is clear in that the single IRB alone is liable for ethical review in multi-institutional non-exempt human participant research. However, it does not prohibit local IRBs from conducting their own review and local IRBs can negotiate to ensure their concerns are addressed. If these negotiations fail, then institutions may instead choose not to participate potentially affecting which institutions contribute to multi-institutional pediatric research. At present, there is no formalized process for resolving such conflicts. Yet, strategies to minimize such nonparticipation are, however, extremely important, as unequal institutional participation in studies has the potential to bias human participant recruitment leading to misleading or even erroneous conclusions. In studies of emergency treatments, failure to effectively reach consensus on exemptions of informed consent can delay crucial advances in the treatment of critically ill patients.

### Institutional differences

Disparities in institutional participation in pediatric research may be exacerbated by the inequalities inherent to hospitals that care for children. Academic pediatric institutions, which currently conduct much existing pediatric research, may have both the resources and experience to be effective single IRBs. However, smaller pediatric hospitals or departments nested within adult hospitals may be less well positioned or resourced to address pediatric IRB issues. This risks that only a select few pediatric institutions will be able to function in the single IRB system, with less resourced groups unable or unwilling to fulfill either the role of relying institution or single IRB. Not only does this threaten to decrease diverse recruitment limiting generalizability, but it could also lead to the overburdening of capable institutions and a greater reliance on commercial IRBs. Critics of IRB commercialization are concerned that the fee-for-service model prioritizes speed over review quality leading to errors, potential adverse consequences, and higher research study costs [3].

### Solutions for implementing single IRBs

Despite such concerns there are examples of successful single IRBs, although often within regional or disease specific consortia [14–16]. Consistency in research topics and participants allows for the development of working relationships and standardized, master RAs that reduce the need for modification and negotiation. The NIH funded “Streamlined, Multisite, Accelerated Resources for Trials” (SMART) IRB aims to replicate this nationally [17]. In addition to online resources, SMART IRB has developed a master RA for its participating institutions. However, how applicable SMART IRB will be to pediatric research and how well a single master RA can account for the wide array of study topics, participants, and local contexts remains to be seen.

The effective and equitable implementation of single IRBs across pediatric institutions will undoubtedly be challenging. However, the CRR demonstrates a determination to improve the multisite research process and an opportunity to shape it for the betterment of investigators and patients. We argue that the same degree of scrutiny and rigor be applied to single IRB implementation as given to other aspects of study conduct. An empiric and collaborative approach among pediatric institutions to develop effective implementation strategies is needed. For example, curated repositories of information about local contexts with details about individual institutional policies and practices could allow single IRBs to proactively prepare study procedures and protocols while expediting RAs. Online applications designed explicitly to facilitate RA negotiations (e.g., Online Reliance System) could aid in task tracking and facilitating information exchange [18]. Outcomes such as time to study initiation, cost, and quality of review should be measured and used for monitoring and comparison with traditional multi-institutional IRB methods. High performing single IRBs and participating institutions should be identified, and lessons learned about barriers and facilitators should be used to optimize the process. Learning collaboratives at both the national and local level would allow for the dissemination of resources, experiences, and innovative ideas that could be adjusted for the diverse types of pediatric institutions. Finally, beginning locally, concerted efforts to harmonize policies and provide direction for pediatric ethical considerations can lay the foundation for wider scalable endeavors.

## Conclusions

In summary, the CRR seeks to improve the efficiency of multisite human participant research in the US. RA negotiations and differences in pediatric specific local contexts and institutions endanger the CRR’s success within pediatric research. These numerous barriers have the potential to limit the willingness of pediatric institutions to be or take part in a single IRB and impede the timely conduct of unbiased clinically relevant research. We argue that an empiric collaborative initiative is needed to optimize implementation of the CRR for pediatric research. If successful, the use of single IRBs has the potential to usher in a new era of high quality impactful multisite pediatric research.

## Data Availability

Not applicable

## Abbreviations

CRR: Common Rule Revision
HRPP: Human Research Participant Protection
IRB: Institutional review board
RA: Reliance agreement
NIH: National Institution of Health
SMART: Streamlined, Multisite, Accelerated Resources for Trials
